# Factitious disorders in Germany–a detailed insight

**DOI:** 10.1007/s12024-021-00395-9

**Published:** 2021-07-02

**Authors:** Julian Prangenberg, Jan Aasly, Elke Doberentz, Burkhard Madea, Harald Schrader

**Affiliations:** 1 Institute of Legal Medicine, University of Bonn, Bonn, Germany; 2 Department of Neuroscience, Faculty of Medicine, Norwegian University of Science and Technology (NTNU), Trondheim, Norway; 3 Department of Neurology, St. Olav’s Hospital, University Hospital of Trondheim, Trondheim, Norway

**Keywords:** Factitious disorders, Munchausen syndrome, Incidence, Germany, Epidemiology

## Abstract

Factitious disorders (FDs) are well known to a majority of physicians; however, the corresponding ICD-10 diagnosis F68.1 remains severely under assigned and often misdiagnosed. Based on a previously conducted nationwide survey in Germany, we extended the analyzed variables to further understand FD characteristics.

The assignments regarding the following variables in the German diagnosis-related group statistics were analyzed: residence of the patient and location of the diagnosing institution, primary referral to the diagnosing institution, reason for admission and discharge, specialty department, total length of stay, length of stay in the longest treating department, surgery performed, case mix revenue, regional type of the treating institution, and patients’ region of origin.

A very distinct difference was observed in the assignment rates based on the homeland of the diagnosed patient and diagnosing institution. The assignment rate showed no significant difference across German regions. Based on our findings, a patient with FD in Germany might exhibit the following “typical” traits: A woman in her late thirties from a rural area is referred by a physician or another hospital wherein she was previously treated for more than a day to an institution for fully inpatient hospital treatment wherein she completes her treatment regularly. Dermatology, neurology, emergency, and internal medicine departments tend to be confronted with patients with FDs more often than other departments; however, surgery is performed in every fifth case. Patients are primarily treated in only one department for ~ 25 days. The case mix revenue will most probably not exceed €5000.

## Introduction

Although Munchausen syndrome [1] and other factitious disorders (FDs) are well known to a majority of physicians, the ICD-10 diagnosis F68.1 is severely under assigned and misdiagnosed. The current Norwegian version of ICD-10, like the English and German versions, defines F68.1 as the “intentional production or feigning of symptoms or disabilities, either physical or psychological”. Moreover, it provides the following description: “The patient feigns symptoms repeatedly for no obvious reason and may even inflict self-harm in order to produce symptoms or signs. The motivation is obscure and presumably internal with the aim of adopting the sick role. The disorder is often combined with marked disorders of personality and relationships”. F68.1 includes “Munchausen syndrome”, “hospital hopper-syndrome”, and “peregrinating patient” and excludes “factitial dermatitis” (L98.1) and “person feigning illness (with obvious motivation)” [2], i.e., Z76.5. One of the reasons for FDs being commonly under assigned and misdiagnosed may be the low awareness of the possibility of this disorder when confronted with an actual patient, fear of stigmatizing the patient with a pejorative connotation, and concerns related to the possibility of reimbursement claims. In some cases, physicians would try to avoid further problems by discharging the patient as quickly as possible. A greater obstacle may be the effort involved in widening the investigation and reviewing previous diagnoses, especially when those have been made in other hospitals. The result is enormous abuse and overuse of healthcare services and, not the least, the risk of irreversible harm to these patients because of unnecessary invasive examinations and repeated interventions. In some cases, the injuries the patients incur as a result of medical treatment are greater than those they inflict on themselves [3–5]. Therefore, FDs may be associated with increased mortality [6]. Moreover, the implementation of unnecessary diagnostic tests and intervention procedures generates significant expenses. In the United States of America, the annual cost was estimated to be $40 million [7].

However, the vast majority of the 1200 PubMed publications on Munchausen syndrome, published after the first description of this disorder by Sir Richard Asher in 1951 [1], are related to clinical presentation. Very few large epidemiological studies have been conducted, in addition to those conducted by Hamilton et al. [8], Schrader et al. [9] and Geile et al. [10]. To further address this issue, we conducted a detailed nationwide epidemiological investigation on the characteristics of the ICD-10 diagnosis F68.1 in general hospitals in Germany.

## Methods

Data from the German Federal Statistical Office (“Statistisches Bundesamt”) (StBA) were used in this study. The StBA is a federal authority of Germany that reports to the Federal Ministry of the Interior. It collects, processes, and analyses statistical information on economics, society, environment, and health from all over Germany. For the present study, the StBA provided data for the annual diagnosis-related group (DRG) statistics. These statistics include every hospital that invoices on the basis of the DRG compensation system, military hospitals that treat civilians, and hospitals of the German employer’s liability insurance association if the casualty or health insurance does not compensate the costs. Prison and police hospitals, as well as psychiatric and psychosomatic institutions, were excluded in these statistics because they use a different accounting system. The DRG statistics were analyzed regarding the assignment of the ICD-10 diagnosis F68.1, residence of the patient and location of the diagnosing institution, primary referral to the diagnosing institution, reason for admission, reason for discharge, grouped specialty department, total length of stay, length of stay in the longest treating department, and surgery performed between 2008 and 2016. Due to adaptions in the DRG statistics during this period, we additionally examined the case mix revenue between 2010 and 2016 as well as the regional type of the treating institution and patients’ region of origin between 2011 and 2016. To comply with the nondisclosure guidelines of the StBA, we had to summarize the specialty departments, the total length of stay, the length of stay in the longest treating department, the case mix revenue, the residence of the patient, and the location of the diagnosing institution. German federal states had to be divided into five regions (six regions for patients’ residence) as some states had too few annual cases. The regions were defined as follows:

Region North: Bremen, Hamburg, Lower Saxony, and Schleswig–Holstein; Region East: Brandenburg, Mecklenburg-Vorpommern, Saxony, Saxony-Anhalt, and Thuringia; Region South: Bavaria and Baden-Württemberg; Region West: North Rhine-Westphalia, Saarland, Rhineland-Palatinate, and Hessen. Region Berlin: Berlin; Others: not reported, unknown, foreign country.

The specialty departments were summarized as follows:

- Specialty department 1: internal medicine, geriatric medicine, cardiology, nephrology, hematology and oncology, endocrinology, gastroenterology, pulmonary disease, and rheumatology.
- Specialty department 2: pediatrics, pediatric cardiology, neonatal-perinatal medicine, and pediatric surgery.
- Specialty department 3: general surgery, orthopedic trauma, neurological surgery, vascular surgery, plastic surgery, thoracic surgery, cardiac surgery, orthopedic surgery, and oral and maxillofacial surgery.
- Specialty department 4: urology, obstetrician/gynecologist care, otorhinolaryngologist, ophthalmology, nuclear medicine, and radiation oncology.
- Specialty department 5: neurology, dermatology, and intensive care.
- Specialty department 6: psychiatry, child and adolescent psychiatry, psychosomatic medicine, and other departments.

An assignment was defined as a single registered hospital case. A diverse gender option for official documents in Germany was introduced at the end of 2018. Hence, no data on diverse genders were available. To calculate the assignment rate per 100,000 inhabitants, we chose the German population of the median year (2012) of the examined period and divided the overall number of assignments by that population.

## Results

**Fig. 1:**
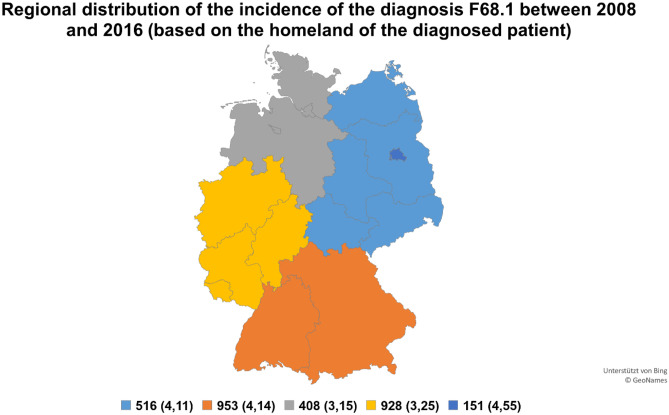
Regional distribution based on the homeland of the diagnosed patient. Total assignments of the diagnosis F68.1 between 2008 and 2016 in defined regions (North, East, West, South, Berlin). The calculated assignment rates per 100,000 inhabitants are indicated in brackets

**Fig. 2:**
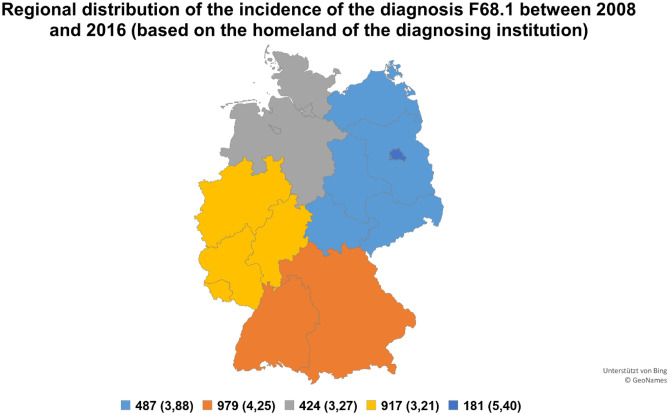
Regional distribution based on the homeland of the diagnosing institution. Total assignments of the diagnosis F68.1 between 2008 and 2016 in defined regions (North, East, West, South, Berlin). The calculated assignment rates per 100,000 inhabitants are indicated in brackets

A total of 2988 assignments with the ICD-10 diagnosis F68.1 were registered from 2008 to 2016 with an annual mean of 332 assignments (ranging between 236 and 465). Germany had approximately 80.5 million inhabitants in 2012 [11], which resulted in a calculated assignment rate of 3.71 per 100,000 inhabitants. A total of 161,527,418 assignments were registered in the DRG statistics during the investigated period, implying that F68.1 was diagnosed in approximately 0.0018% of all assignments. A considerable gender difference was noticed, with 63% female and 37% male assignments, and a difference was also observed regarding the mean age of those two genders (weighted mean of 38.1 and 41.4 years, respectively). The weighted arithmetic mean age for all assignments and genders was 39.3 years [10]. The reason for admission was almost entirely a fully inpatient hospital treatment (2883 assignments, 96.5%), followed by a fully inpatient hospital treatment with pre-stationary treatment (97 assignments, 3.2%) and stationary childbirth (8 assignments, 0.3%). The mean number of treating departments was 1.35. The total length of stay varied between 0 and 25 days in the vast majority of the cases (2717 assignments, 90.9%), 177 assignments (5.9%) had a hospital stay between 25 and 50 days and only 94 assignments (3.1%) had a stay longer than 50 days. The distribution of the length of stay was almost identical in the longest treating department: 2734 assignments (91.5%) were identified with a stay between 0 and 25 days, 163 assignments between 25 and 50 days (5.5%) and only 91 assignments (3.0%) with a stay longer than 50 days. Surgery was performed in about every fifth case (628 assignments, 21.0%). The results of the other analyzed variables are shown in Figs. 1 and 2 and Tables 1, 2, 3, 4, 5, 6.

**Table 1:** Primary referral to the institution that assigned the diagnosis F68.1

Primary referral to the diagnosing institution	Assignments	Percentage (%)
Transfer from another hospital with previous treatment longer than 24 h	1539	51.5
Physician	1170	39.2
Transfer from another hospital with previous treatment up to 24 h	234	7.8
Emergency	44	1.5
Missing information	1	0.0
Total	2988	100.0

**Table 2:** Reason for discharge

Reason for discharge	Assignments	Percentage (%)
Treatment regularly completed	2090	69.9
Transfer to a different hospital	335	11.2
Treatment ended against medical advice	228	7.6
Treatment regularly completed, aftercare treatment planned	141	4.7
Internal transfer with change between the compensation systems, change between fully and partially inpatient treatment	61	2.0
Discharge to a care facility	38	1.3
External transfer for psychiatric treatment	35	1.2
Treatment completed due to other reasons	27	0.9
Discharge to a rehabilitation facility	21	0.7
Treatment ended against medical advice, aftercare treatment planned	6	0.2
Death	6	0.2
Total	2988	100.0

**Table 3:** Distribution between the grouped specialty departments

Specialty department groups	Assignments	Percentage (%)
Specialty department 1	1255	42.0
Specialty department 2	286	9.6
Specialty department 3	734	24.6
Specialty department 4	179	6.0
Specialty department 5	502	16.8
Specialty department 6	32	1.1
Total	2988	100.0

**Table 4:** Case Mix revenue

Case Mix Revenue (between 2010 and 2016)	Assignments	Percentage (%)
0 to 5000 Euro	1763	83.4
5000 to 10,000 Euro	182	8.6
10,000 Euro and more	119	5.6
Missing information	51	2.4
Total	2115	100.0

**Table 5:** Regional type of the institution that assigned the diagnosis F68.1

Regional type of treating institution (between 2011 and 2016)	Assignments	Percentage (%)
Urban area	807	46.2
Area with beginning urbanization	546	31.3
Rural area	392	22.5
Total	1745	100.0

**Table 6:** Patients’ region of origin

Patients’ region of origin (between 2011 and 2016)	Assignments	Percentage (%)
Unknown	20	1.1
Urban area	694	39.8
Area with beginning urbanization	559	32.0
Rural area	472	27.0
Total	1745	100.0

## Discussion

There is a relative scarcity of systematic studies on the incidence of FDs, with the major obstacle of obtaining a reliable and valid incidence being the nature of this disorder itself. The current data primarily consist of case reports and single-case studies [12]. To the best of our knowledge, this is the first detailed national survey regarding the incidence of diagnosis of FDs in general hospitals in Germany.

Patients with FDs are primarily referred to the medical institutions by physicians or another hospital wherein the patients were treated for at least 24 h for an inpatient hospital stay. It remains unclear whether in the eight cases with stationary childbirth the patients presented symptoms that led to the suspicion of an emerging childbirth or whether a Munchausen syndrome by proxy was the reason for assigning the diagnosis F68.1. Considering the nature of FDs, it is not surprising that a certain proportion of the patients discharged themselves against medical advice, despite the majority of patients completing the treatment regularly.

The potential harm that patients with FDs might inflict on themselves is supported by the fact that surgery is performed in almost every fifth case and that the primary contact with a healthcare institution of a small proportion of patients is an emergency admission. Moreover, six patients died during their hospital stay, although no information could be collected regarding the cause of death and the specific circumstances.

The actual incidence and distribution among the different medical specialties still remains unknown. To comply with the nondisclosure guidelines of the StBA, the medical departments had to be summarized. Hence, it was not possible to narrow down single departments that really diagnose F68.1 more often than others. Considering the size of the summarized departments (neurology, dermatology, and intensive care), it can be assumed that these departments are relatively often confronted with patients with FDs. This would also be primarily in accordance with the reviews published by Yates et al. and Caselli et al., who concluded that patients with FDs are, among other departments, most likely seen in dermatology, neurology, emergency, and internal medicine departments [13, 14].

The calculated assignment rate per 100,000 inhabitants was slightly higher in Berlin, Eastern Germany, and Southern Germany than in Northern and Western Germany (Figs. 1 and 2). Only a very distinct difference was observed in the assignment rates based on the homeland of the diagnosed patient and diagnosing institution. This may indicate that patients with FDs do not visit institutions far away from their home.

Based on a nationwide survey conducted in 2011 on the degree of urbanization, approximately 40% of people in Germany live in urban areas and areas with the beginnings of urbanization, respectively, and approximately 40% of them live in rural areas [15]. These results may indicate that FDs are slightly more frequent in rural areas and slightly less common in areas with the beginnings of urbanization. In addition, it can be predicted that patients tend to visit institutions in urban areas.

In conclusion, a patient with FD in Germany might show the following “typical” traits: A woman in her late thirties from a rural area is referred by a physician or another hospital in which she was previously treated for more than a day to an institution for fully inpatient hospital treatment wherein she completes her treatment regularly. Dermatology, neurology, emergency, and internal medicine departments tend to be more often confronted with patients with FDs than other departments; however, surgery is performed in every fifth case. The patient is primarily treated in only one department for up to 25 days. The case mix revenue will most probably not exceed €5000.

Due to strict German medical confidentiality laws, some variables had to be summarized. Therefore, certain issues concerning FDs could only be vaguely answered at best. This remains a major limitation in breaking down the aspects of FDs. Nonetheless, the presented data on the nationwide survey provide a first insight into the nature of FDs. It appears advisable to perform such surveys in other countries to gain a better insight into the characteristics of FDs.

## Key points

1. The regional frequency of factitious disorders does not differ significantly across Germany.
2. Surgery is performed in every fifth case.
3. Patients with FDs are primarily treated in only one department.
4. Treatment generally lasts no longer than 25 days.
5. The case mix revenue mostly does not exceed €5000.
